# The serotonin 6 receptor controls neuronal migration during corticogenesis via a ligand-independent Cdk5-dependent mechanism

**DOI:** 10.1242/dev.108043

**Published:** 2014-09

**Authors:** Moritz Jacobshagen, Mathieu Niquille, Séverine Chaumont-Dubel, Philippe Marin, Alexandre Dayer

**Affiliations:** 1 Department of Psychiatry, University of Geneva Medical School, CH-1211 Geneva 4, Switzerland; 2 Department of Basic Neurosciences, University of Geneva Medical School, CH-1211 Geneva 4, Switzerland; 3 Institut de Génomique Fonctionnelle, CNRS UMR 5203, INSERM U661, Universités Montpellier I & II, Montpellier 34094, France

**Keywords:** 5-HT6 receptor, Migration, Serotonin, Cortex, Cdk5, Mouse

## Abstract

The formation of a laminar structure such as the mammalian neocortex relies on the coordinated migration of different subtypes of excitatory pyramidal neurons in specific layers. Cyclin-dependent kinase 5 (Cdk5) is a master regulator of pyramidal neuron migration. Recently, we have shown that Cdk5 binds to the serotonin 6 receptor (5-HT6R), a G protein-coupled receptor (GPCR). Here, we investigated the role of 5-HT6R in the positioning and migration of pyramidal neurons during mouse corticogenesis. We report that constitutive expression of 5-HT6R controls pyramidal neuron migration through an agonist-independent mechanism that requires Cdk5 activity. These data provide the first *in vivo* evidence of a role for constitutive activity at a GPCR in neocortical radial migration.

## INTRODUCTION

Cortical circuit formation relies on the migration of pyramidal neurons (PNs) into specific layers (Rakic et al., 2007; Marin et al., 2010) and cell-extrinsic factors such as neurotransmitters have been shown to regulate their migration (Heng et al., 2007). Serotonin is detected early in the developing mouse embryonic telencephalon (Bonnin et al., 2011) and regulates a variety of cellular processes involved in cortical circuit formation, including neuronal migration (Gaspar et al., 2003; Riccio et al., 2009, 2011; Vitalis et al., 2013). Distinct steps are involved in the migration of PNs before they reach their final position in the cortical plate (CP) (Heng et al., 2007; Rakic et al., 2007; Marin et al., 2010). After being generated from radial glia in the ventricular zone (VZ), late-born PNs migrate a short distance to reach the subventricular zone (SVZ) and intermediate zone (IZ) where they transiently adopt a multipolar morphology before ascending radially towards the CP (Nadarajah and Parnavelas, 2002; Noctor et al., 2004). Serine/threonine cyclin-dependent kinase 5 (Cdk5) has been shown to be a master regulator of PN migration (Su and Tsai, 2011). Genetic loss-of-function experiments demonstrate that Cdk5 (Ohshima et al., 1996, 2007; Gilmore et al., 1998) and its activator p35 (Chae et al., 1997; Kwon and Tsai, 1998) are required *in vivo* for the proper radial migration of pyramidal neurons. Recently, we have shown that the serotonin 6 receptor (5-HT6R) binds to Cdk5 (Meffre et al., 2012). Here, we have investigated *in vivo* the role of the 5-HT6R on the migration of PNs using cell-type-specific genetic approaches.

## RESULTS

**Fig. 1.**
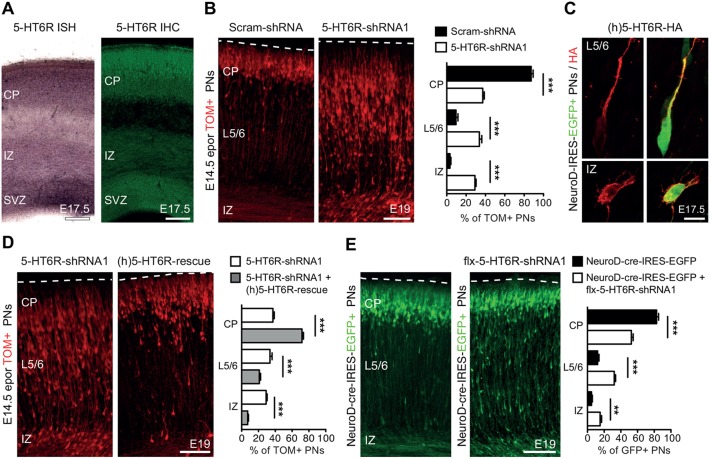
***In vivo* downregulation of 5-HT6R induces mispositioning of superficial layer pyramidal neurons.** (A) *In situ* hybridization (ISH) and immunohistochemistry (IHC) showing that 5-HT6R is expressed in the SVZ, IZ and CP of the developing cortex. (B) *In utero* electroporation of 5-HT6R-shRNA1 carried out at E14.5 induces a significant mispositioning of TOM+ PNs in the IZ, layer 5/6 and CP compared with scram-shRNA at E19. (C) *In utero* electroporation of HA-tagged (h)5-HT6R-rescue plasmid, together with NeuroD-IRES-EGFP was carried out at E14.5. IHC for HA shows expression of the 5-HT6R at the membrane of GFP+ multipolar PNs in IZ and bipolar PNs migrating towards the pial surface. (D) E14.5 *in utero* electroporation of a (h)5-HT6R-rescue plasmid significantly rescues the 5-HT6R-shRNA1-induced mispositioning of PNs. (E) *In utero* electroporation of a NeuroD-cre-IRES-EGFP plasmid with a lox flanked (flx)-5HT6R-shRNA1 construct phenocopies the significant mispositioning of PNs in the IZ, layer 5/6 and CP. PNs, pyramidal neurons; SVZ, subventricular zone; IZ, intermediate zone; CP, cortical plate. ***P<0.001, **P<0.01, unpaired Student's *t*-test. Data are mean±s.e.m. Scale bars: 100 µm in A,B,D,E; 10 µm in C.

*In situ* hybridization (ISH) and immunohistochemistry (IHC) revealed that 5-HT6R is expressed in the SVZ, IZ and CP of the developing embryonic pallium from E14.5 to E17.5 (Fig. 1A; supplementary material Fig. S1A). *In utero* electroporation at E14.5 in the lateral ventricular zone (VZ) of the pallium was used to label PNs migrating to superficial cortical layers. IHC indicated that 5-HT6R was expressed in electroporated PNs, suggesting that it could regulate their migration (supplementary material Fig. S1B). To test this hypothesis, *in utero* electroporation of a shRNA plasmid targeting the *Htr6* gene (5-HT6R-shRNA1) or a scrambled shRNA (scram-shRNA) was performed at E14.5 and brains were assessed at subsequent developmental time-points. Analysis of electroporated brains at E19.0 revealed that 5-HT6R-shRNA1 tdTomato-labelled (TOM+) PNs were significantly misplaced in the CP, in deep cortical layers and in the IZ compared with scram-shRNA-treated PNs (Fig. 1B). 5-HT6R-shRNA1 efficiently downregulated 5-HT6R-EGFP expression *in vivo* and *in vitro* (supplementary material Fig. S2A-D). A second shRNA targeting 5-HT6R induced a similar mispositioning phenotype (supplementary material Fig. S2E). To determine whether the 5-HT6R-shRNA1-induced mispositioning phenotype was specific to 5-HT6R downregulation, a HA-tagged human (h)5-HT6R plasmid containing three silent mutations in the 5-HT6R-shRNA1 recognition binding site was used for rescue experiments. Immunohistochemistry revealed hemagglutinin (HA)+ membrane expression of the 5-HT6R in multipolar pyramidal neuron progenitors in IZ and in bipolar PNs migrating towards the pial surface (Fig. 1C). Overexpression of the (h)5-HT6R construct induced only minor effects on PN positioning (supplementary material Fig. S2F) but significantly rescued the mispositioning of 5-HT6R-shRNA1 PNs (Fig. 1D).

The 5-HT6R-shRNA1 induced a migratory phenotype without affecting earlier steps of progenitor cell proliferation or differentiation. Indeed, 5-HT6R-shRNA1 mispositioning was induced using a cre/lox system allowing conditional knockdown of 5-HT6R in postmitotic migratory neurons (Fig. 1E). The fraction of 5-HT6R-shRNA1 PNs reaching the CP using this cre/lox strategy was higher compared with the non-conditional approach. This could be due to the fact that NeuroD-cre mediated recombination produces a temporal delay in the expression of high levels of 5-HT6R-shRNA, leading to a less efficient 5-HT6R knockdown in migrating PNs and, thus, decreased mispositioning compared with the non-conditional approach. 5-HT6R knockdown did not affect proliferation and neuronal differentiation. Indeed, a BrdU proliferation index at E15.5 revealed no significant differences in the fraction of BrdU+ 5-HT6R-shRNA1 TOM+ progenitors compared with scram-shRNA TOM+ controls (supplementary material Fig. S3A). At E15.5, the fraction of electroporated TOM+ cells expressing TBR2 or NGN2, two key regulators of early neuronal differentiation (Greig et al., 2013), was not modified following 5-HT6R knockdown (supplementary material Fig. S3B,C). At E17.5, the proportion of TOM+ cells expressing the POU-III transcription factor BRN2, a key regulator of upper-layer PN differentiation (Dominguez et al., 2013), was likewise unchanged (supplementary material Fig. S3D). Finally, the fraction of TOM+ neurons expressing SATB2, a transcription factor controlling upper-layer molecular identity (Greig et al., 2013), and TBR1, a transcription factor controlling lower-layer molecular identity (Greig et al., 2013), was not affected by 5-HT6R knockdown (supplementary material Fig. S3E,F).

**Fig. 2.**
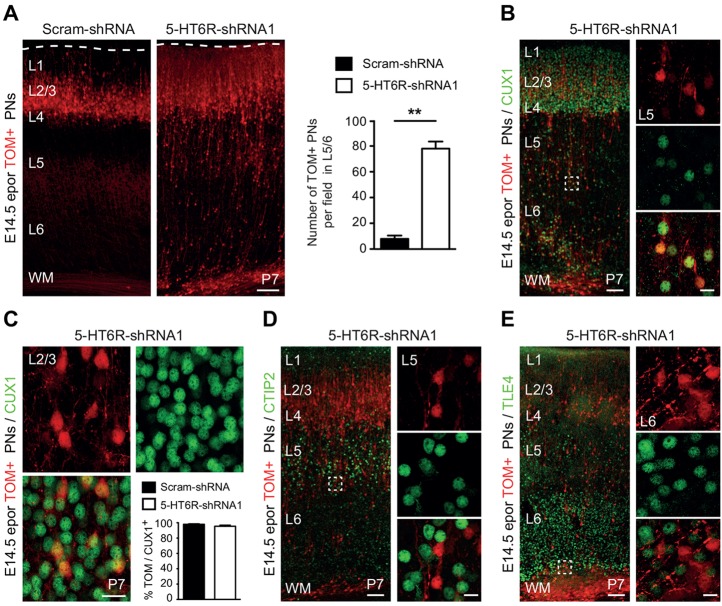
***In vivo* downregulation of 5-HT6R induces persistent mispositioning of pyramidal neurons that maintain their superficial layer molecular identity.** (A) *In utero* electroporation at E14.5 leads to a persistent mispositioning of 5-HT6R-shRNA1 TOM+ PNs at P7 in WM and deep cortical layers compared with scram-shRNA (**P<0.01, unpaired Student's *t*-test). (B,C) Immunohistochemistry (IHC) for CUX1 shows that mispositioned 5-HT6R-shRNA1 TOM+ PNs in deep cortical layers maintain a superficial layer molecular identity (B) and that the fraction of TOM+ PNs expressing CUX1 in cortical layers 2/3 is unchanged after 5-HT6R knockdown (C). (D,E) IHC showing that misplaced 5-HT6R-shRNA1 TOM+ PNs are not immunolabelled for CTIP2 (D) specifically expressed in layer 5 subcerebral projection neurons or for TLE4 (E) expressed in layer 6 thalamo-cortical projection neurons. PNs, pyramidal neurons; CUX1, Cut-like homeobox 1; CTIP2, COUP-TF-interacting protein 2; TLE4, transducin-like enhancer of split 4; WM, white matter. Data are mean±s.e.m. Scale bars: 100 µm in A and in low magnification views in B,D,E; 20 µm in C; 15 µm in high magnification views in B,D,E.

*In utero* electroporation of 5-HT6R-shRNA1 induced a persistent mispositioning of PNs in the postnatal cortex. At P7, 5-HT6R-shRNA1 TOM+ PNs were significantly misplaced in deep cortical layers and in the underlying white matter (Fig. 2A). Displaced TOM+ cells found in deep cortical layers maintained their molecular identity of superficial layer PNs as a majority of them (72.1±4.9%) expressed the superficial layer-specific transcription factor CUX1 (Fig. 2B) (Greig et al., 2013). The percentage of TOM+ PNs expressing CUX1 in upper layers was not modified following 5-HT6R knockdown (Fig. 2C). Moreover, displaced TOM+ cells did not express the layer 5-specific transcription factor CTIP2 (Fig. 2D) or the layer 6-specific transcription factor TLE4 (Fig. 2E) (Greig et al., 2013).

**Fig. 3.**
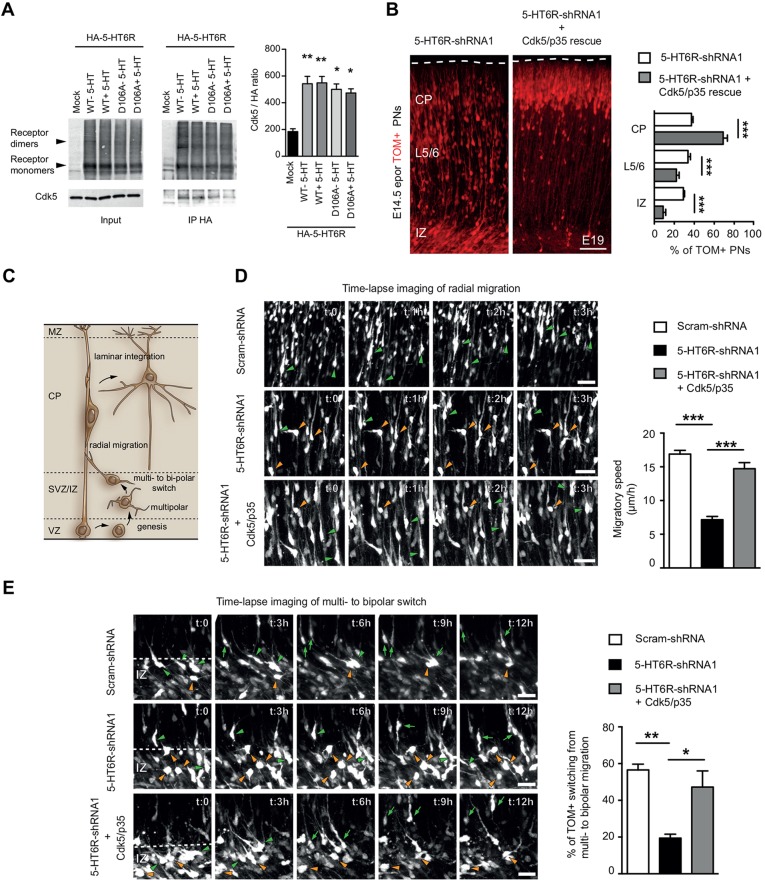
**5-HT6R controls PN migration through a Cdk5-dependent mechanism.** (A) NG108-15 cells were transfected with empty plasmid (mock), with HA-tagged (h)5-HT6R (WT) or a 5-HT6R construct mutated on the serotonin-binding site (D106A) and exposed or not to serotonin (5-HT) (1 µM). The inputs correspond to 5% of the total amount of protein used for immunoprecipitation. Co-immunoprecipitated Cdk5 was detected after HA immunoprecipitation. Quantification of immunoprecipitated Cdk5 indicates a significant increase in the wild-type and D106A conditions compared with the mock control with no effects of serotonin. Data, expressed in arbitrary units, were calculated as ratios of Cdk5 to HA immunoreactive signals in immunoprecipitates. Data are mean±s.e.m. of values obtained in four independent experiments (**P<0.01, *P<0.05, one-way ANOVA, Tukey's post hoc test). (B) *In utero* electroporation of Cdk5/p35 significantly rescues 5-HT6R-shRNA1-induced mispositioning of PNs compared with scram-shRNA (***P<0.001, unpaired Student's *t*-test). (C) Two distinct steps in the migration of PNs: the switch from a multi- to bipolar radial migration and radial glial-guided locomotion. (D) Time-lapse imaging showing that locomotion speed of 5-HT6R-shRNA1 TOM+ PNs through deep layers is significantly decreased compared with scram-shRNA and significantly rescued by Cdk5/p35 electroporation. Orange arrowheads indicate cells that remain stationary during the time-lapse sequence, whereas green arrowheads indicate cells that migrate radially. (E) Time-lapse imaging showing that the percentage of 5-HT6R-shRNA1 TOM+ PNs switching from multipolar stage (green arrowheads) to radial migration (green arrows) is significantly decreased versus scram-shRNA and significantly rescued by Cdk5/p35 electroporation. Orange arrowheads indicate multipolar cells that do not switch to radial migration during the time-lapse sequence (***P<0.001, **P<0.01, *P<0.05 one-way ANOVA, Tukey's post hoc test). PNs, pyramidal neurons; MZ, marginal zone; CP, cortical plate; SVZ, subventricular zone; IZ, intermediate zone. Data are mean±s.e.m. Scale bars: 100 µm in B; 40 µm in D; 25 µm in E.

**Fig. 4.**
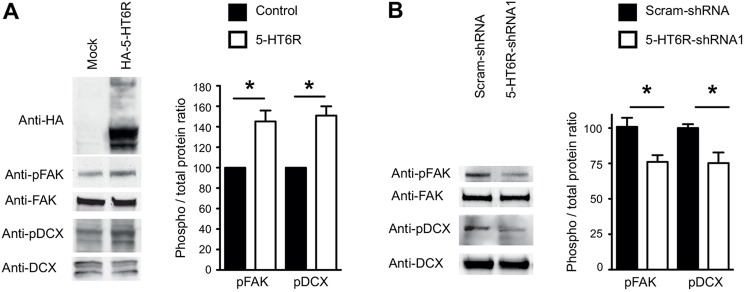
**5-HT6R regulates activity of Cdk5 substrates that control neuronal migration.** (A) NG108-15 cells were transfected with empty plasmid (mock) or with a plasmid encoding an HA-tagged (h)5-HT6R. Quantification of western blots showed an increase in the phosphorylation of focal adhesion kinase (pFAK on S732) and doublecortin (pDCX on S297) in cells transfected with the 5-HT6R compared with the mock control (*P<0.05, unpaired Student's *t*-test). Data, expressed as a percentage of values measured in Mock cells, are mean±s.e.m. of values obtained in three independent experiments. (B) E14.5 cortical cultures were nucleofected with 5-HT6R-shRNA1 and scram-shRNA, and western blots were performed on cell lysates at 3 days *in vitro* (DIV3). Quantification of western blots revealed a decrease is pFAK and pDCX in cells nucleofected with the 5-HT6R-shRNA1 compared with the scram-shRNA condition (*P<0.05, unpaired Student's *t*-test). Data are mean±s.e.m. of values obtained in a least three independent experiments.

In a recent proteomic study, we found that the 5-HT6R binds to Cdk5 in HEK-293 cells (Meffre et al., 2012). To confirm this interaction, we performed co-immunoprecipitation (co-IP) experiments in the neuroblastoma-glioma NG108-15 cells and found that Cdk5 is associated with the HA-tagged 5-HT6R (Fig. 3A). To determine whether 5-HT6R-shRNA1 affects migration of PNs through a Cdk5-dependent mechanism, we used an *in vivo* rescue strategy. Combined electroporation of plasmids expressing Cdk5 and its activator p35 led to a significant rescue of the 5-HT6R-shRNA1-induced mispositioning phenotype (Fig. 3B), indicating that the 5-HT6R regulates migration of PNs via a Cdk5-dependent mechanism. Previous studies have shown that Cdk5 regulates two distinct steps in the migration of PNs: the transition from a multipolar to a migratory bipolar morphology and the process of radial glial-guided locomotion (Fig. 3C) (Ohshima et al., 2007; Nishimura et al., 2010). To directly visualize the effect of the 5-HT6R-shRNA1 on the different steps of the migratory process, we performed confocal time-lapse recordings on cortical slices. Quantification revealed that in deep cortical layers the migratory speed of radially migrating 5-HT6R-shRNA1 TOM+ cells was significantly decreased versus scram-shRNA (Fig. 3D; supplementary material Movies 1 and 2). In the IZ, the proportion of 5-HT6R-shRNA1 TOM+ PNs switching from a multipolar to a bipolar migratory morphology was significantly decreased when compared with scram-shRNA (Fig. 3E; supplementary material Movies 3 and 4). Strikingly, Cdk5/p35 overexpression significantly rescued the defects induced by 5-HT6R knockdown in these two distinct migratory steps (Fig. 3D,E; supplementary material Movies 5 and 6). Finally, to determine whether 5-HT6R and Cdk5 interact functionally, we assessed Cdk5 substrates controlling PN migration, such as doublecortin (DCX) and the focal adhesion kinase (FAK) (Tanaka et al., 2004; Xie et al., 2003). Expression of (h)5-HT6R significantly increased phosphorylation of DCX at serine 297 and FAK at serine 732 in NG108-15 cells, whereas 5-HT6R knockdown in embryonic primary cortical cultures significantly reduced phosphorylation of DCX and FAK (Fig. 4A,B). Taken together, these data indicate that the 5-HT6R regulates the phosphorylation of Cdk5 substrates controlling neuronal migration.

**Fig. 5.**
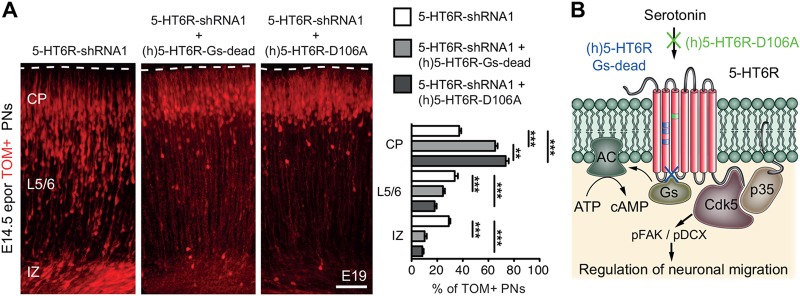
**5-HT6R controls PN migration through an agonist-independent mechanism.** (A) *In utero* electroporation of (h)5-HT6R-Gs dead or (h)5-HT6R-D106A significantly rescues 5-HT6R-shRNA1-induced mispositioning of PNs compared with scram-shRNA (***P<0.001, **P<0.01, one-way ANOVA, Tukey's post hoc test). Data are mean±s.e.m. Scale bars: 100 µm in A. (B) 5-HT6R signalling pathways and their role in migration of PNs. Cdk5/p35 overexpression, 5-HT6R plasmids abolishing serotonin-induced cAMP signalling [(h)5-HT6R-D106A] and Gs-dependent cAMP signalling [(h)5-HT6R-Gs-dead] rescue the 5-HT6R-shRNA1-induced mispositioning phenotype. PNs, pyramidal neurons; IZ, intermediate zone; CP, cortical plate; AC, adenylyl cyclase.

The 5-HT6R is a G protein-coupled receptor (GPCR) positively coupled to adenylyl cyclase, which exhibits high constitutive activity (Kohen et al., 2001). To determine whether constitutive or serotonin-elicited cAMP signalling are required for 5-HT6R-dependent migration, rescue experiments were performed using a (h)5-HT6R-Gs-dead mutant, in which constitutive and serotonin-induced cAMP signalling are abolished, or a (h)5-HT6R-D106A mutant, in which only serotonin-induced cAMP activation is abolished (Fig. 5; supplementary material Fig. S4). Strikingly, both mutants significantly rescued 5-HT6R-shRNA1-induced mispositioning of PNs (Fig. 5), indicating that constitutive and agonist-dependent Gs signalling are not required for 5-HT6R-operated regulation of migration. In addition, co-IP experiments indicated that addition of serotonin did not modify the association of Cdk5 with the 5-HT6R (Fig. 3A), further supporting the theory that serotonin activation is not a major regulator of this interaction.

## DISCUSSION

Taken together, our results demonstrate that the 5-HT6R controls migration of PNs through an agonist-independent, Cdk5-dependent, mechanism. These results are concordant with the fact that layer-specific positioning of PNs is not altered by serotonin depletion *in vivo* (Narboux-Neme et al., 2013). However, it should be mentioned that an excess of serotonin, as well as pharmacological manipulation of 5-HT6R, have been shown to affect neuronal migration in cortical slices (Riccio et al., 2009, 2011), suggesting a role for ligand-induced activation of the 5-HT6R in modulating neuronal migration, possibly in conditions where there is a pathological excess of serotonin. Furthermore, altered embryonic positioning of PNs is observed *in vivo* in serotonin transporter homozygous knockout mice, suggesting that a pathological excess of serotonin can modify the migration of PNs (Riccio et al., 2011). Whether this phenotype is 5-HT6R dependent remains to be determined. Interestingly, in another cellular process involved in cortical circuit formation, thalamocortical axon segregation in the barrel cortex, an excess of serotonin has consistently been shown to have an effect (Persico et al., 2001; Cases et al., 1996); barrel cortex formation is normal in serotonin depletion models *in vivo* (Narboux-Neme et al., 2013). The migratory defects induced by 5-HT6R downregulation partially phenocopy the Cdk5 loss-of-function phenotypes previously reported in the literature (Ohshima et al., 1996, 2007; Gilmore et al., 1998). The observations that 5-HT6R binds to Cdk5 and regulates the phosphorylation of Cdk5 substrates (such as DCX and FAK), and that Cdk5/p35 rescues the 5-HT6R knockdown migratory phenotype, strongly support the possibility that 5-HT6R is an upstream membrane regulator of Cdk5 activity. Proper targeting and activity of Cdk5 at the plasma membrane is determined by N-terminal myristoylation and phosphorylation of p35 (Asada et al., 2012; Patrick et al., 1999). Expression of 5-HT6R at the plasma membrane could thus provide an additional mechanism that controls the activity of Cdk5 during neuronal migration. Further work will be necessary to determine the precise structural and functional interactions between Cdk5/p35 and 5-HT6R during neuronal migration.

## MATERIALS AND METHODS

### *In utero* electroporation and plasmids

Animal experiments were conducted according to Swiss and international guidelines, and approved by the local Geneva animal care committee. Embryos from time-mated pregnant E14.5 C57-BL6 mice were electroporated in the lateral VZ of the dorsal pallium as described previously (Riccio et al., 2011). The following plasmids were used at concentrations of 0.75 µg/ml or 1 µg/ml and were co-electroporated in equal ratios in control and experimental conditions: 5-HT6R-shRNA1 (TRCN0000027429 mature sense: GCGCAACACGTCTAACTTCTT, Thermoscientific), 5-HT6R2-shRNA (TRCN0000027469 mature sense: GCCATGCTGAACGCGCTGTAT, Thermoscientific) and scrambled shRNA (mature sense: CCTAAGGTTAAGTCGCCCTCG, Addgene), which were under the regulation of the human U6 promoter; pUB6-tdTomato, pUB6-Cdk5, pUB6-p35, pUB6 human (h)5-HT6R containing three silent mutations in the 5-HT6R-shRNA1 binding region [(h)5-HT6R-rescue], pUB6 (h)5-HT6R-rescue backbone containing three mutations (F69L, T70I, D72A) at conserved transmembrane domain II residues (which abolished constitutive and serotonin-induced cAMP signalling through Gs) [(h)5-HT6R-Gs-dead] (Harris et al., 2010) and pUB6 (h)5-HT6R-rescue backbone containing a D106A mutation (which abolished serotonin-induced cAMP signalling) [(h)5-HT6R-D106A] (Zhang et al., 2006), which were under the regulation of the ubiquitin promoter (pUB6). The pUB6 (h)5-HT6R-rescue backbone contained an N-terminal HA tag. In addition, we used a mouse (m)5-HT6R with an N-terminal EGFP tag under the regulation of the CMV promoter (pCMV) [(m)5-HT6R-EGFP) (a kind gift from K. Mykytyn, Ohio State University, USA), pCAG-GFP (Addgene) and a NeuroD-IRES-EGFP (a kind gift from L. Nguyen, University of Liège, Belgium) (Hand et al., 2005). For conditional shRNA experiments using the cre-lox system, a NeuroD-Cre-IRES-EGFP (a kind gift from L. Nguyen) was co-electroporated with a floxed 5-HT6R-shRNA1 cloned in the pSico construct (Addgene) (Ventura et al., 2004).

### Tissue processing and immunohistochemistry

Pregnant females or postnatal animals were euthanized by lethal intreperitoneal injection of pentobarbital (50 mg/kg). Brains from embryos were dissected and fixed overnight (O.N.) in cold paraformaldehyde (PFA, 4%, pH 7.4). For postnatal brains, animals were perfused with intracardial 0.9% saline followed by cold 4% PFA. Brains were cut on a Vibratome (Leica VT100S) for immunohistochemistry (IHC). Sections were kept at 4°C in 0.1 M phosphate buffer saline (PBS) and were stained as described (Riccio et al., 2011) with the following primary antibodies: goat anti-GFP (1/500, Abcam, ab5450), goat anti-GFP (1/1000, Millipore, AB3080), rabbit anti-5-HT6R (1/500, Abcam, ab103016), rabbit anti-CUX1 (1/250, Santa Cruz, sc-13024), rabbit anti-TLE4 (1/500, Santa Cruz, sc-9125), rat anti-CTIP2 (1/500, Abcam, ab18465), rabbit anti-TBR2 (1/500, Abcam, ab23345), rabbit anti-TBR1 (1/500, Abcam, ab31940), goat anti-Ngn2 (1/50, Santa-Cruz, sc-19233), goat anti-Brn2 (1/50, Santa-Cruz, sc-6029), mouse anti-SATB2 (1/500, Abcam, ab51502), secondary goat or donkey Alexa-488, -568 and -647 antibodies (Molecular Probes, Invitrogen) raised against the appropriate species were used at a dilution of 1/1000 and sections were counterstained with Hoechst 33258 (1/10,000, Life Technologies, H3569).

### *In situ* hybridization

Sections were hybridized as described previously (Riccio et al., 2011). The antisense 5-HT6R digoxigenin-labelled RNA probe was synthesized by *in vitro* transcription using a DIG RNA labelling kit and T7 RNA polymerase (Roche). The forward primer: 5′-TCCAGGTCTCTTCGATGTCC-3′ and reverse primer: 5′-CGATGTTAATACGACTCACTATAGGGCCGTATC-TCAGGCTCCACAG-3′ (underlined section indicates the T7 promoter and linker sequence) were designed in exon 4 of the 5-HT6R gene. The unbound probe was washed and slices were incubated with alkaline phosphatase-conjugated anti-DIG antibody (1/2000, Roche, #11093274910) overnight at 4°C. NBT/BCIP was then used as an alkaline substrate to reveal the hybridized probe.

### Cell cultures and transfection

HEK-293 cells were transfected with pCMV-5-HT6R-EGFP and 5-HT6R-shRNA1 or scram-shRNA using TurboFect (Thermoscientific) or Lipofectamine 2000 (Life Technologies) and maintained in DMEM supplemented with 10% foetal calf serum and penicillin-streptomycin (Invitrogen) under standard conditions (37°C, 5% CO_2_). NG108-15 cells were transfected using Lipofectamine 2000 (Invitrogen) with either empty plasmid or with a plasmid encoding the HA-tagged (h)5-HT6R and grown for 24 hours in DMEM supplemented with 10% foetal calf serum and HAT supplement (Life Technologies). Primary neuronal cultures were prepared as previously described (Rice et al., 2010; Riccio et al., 2011). Briefly, E14.5 cortices or E17.5 cortices previously electroporated at E14.5 to label PNs were dissected in ice-cold HBSS (Life Technologies), trypsinized (0.25% Trypsin-EDTA, Life Technologies) for 20 min at 37°C and 5% CO_2_, centrifuged for 5 min at 1200 rpm (124 ***g***) and resuspended in neurobasal medium (NBM). For 5-HT6R knockdown experiments, ∼2×10^6^ cells/well were nucleofected with either 5 µg scrambled shRNA or 5 µg 5-HT6R-shRNA1, according to the Amaxa Mouse Neuron Nucleofector Kit (Lonza). Cells were seeded onto six-well plates coated with 0.25 µg/µl poly-D-lysine (Sigma) supplemented with NBM and maintained in culture at 37°C and 5% CO_2_.

### Western blotting

Twenty-four hours after transfection, cells were lysed in a solubilization buffer containing HEPES 20 mM (pH 7.4), 150 mM NaCl, 1% NP40, 10% glycerol, 4 mg/ml dodecylmaltoside, phosphatase inhibitors (NaF, 10 mM; sodium vanadate, 2 mM; sodium pyrophosphate, 1 mM; β-glycerophosphate, 50 mM) and a protease inhibitor cocktail (Roche) for 1 h at 4°C. Proteins were resolved on 10% polyacrylamide gels and transferred onto Hybond C nitrocellulose membranes (GE Healthcare). Membranes were immunoblotted with primary antibodies: rabbit anti-GFP (1/1000, Millipore, AB3080), mouse anti-GAPDH (1/1000, Cell Signaling, #2118), rabbit anti-phosphoFAK (S732) (1/1000, Abcam, ab4792), rabbit anti-FAK (1/500, Cell Signaling, #3285), rabbit anti-phosphoDCX (Ser297) (1/1000, Cell Signaling, #4605), rabbit anti-DCX (1/500, Cell Signaling, #4604), mouse anti-HA (1/1000, Covance, MMS-101R), rabbit anti-GAPDH (1/1000, Santa-Cruz, sc-25778), rabbit anti-β-tubulin (1/1000, Abcam, ab6046) then with horseradish peroxidase-conjugated anti-mouse Fab or anti-rabbit secondary antibody (both at 1/5000, Jackson ImmunoResearch and GE Healthcare, 115-035-174 and NA934). Immunoreactivity was detected with an enhanced chemiluminescence method (ECL plus detection reagent, GE Healthcare) and immunoreactive bands were quantified by densitometry using the ImageJ software in three independent experiments.

### Co-immunoprecipitation experiments

NG108-15 cells were transfected with either empty plasmid or with plasmids encoding the HA-tagged wild-type 5-HT6R or the (h)5-HT6R-D106A construct mutated on the serotonin-binding site. Transfected cells were plated on two plates, only one of which was treated with serotonin (1 µM), 4 h prior to cell lysis. After 24 h, cells were lysed as described above. Solubilized proteins (1 mg per condition) were incubated with agarose-conjugated anti-HA antibody (Sigma-Aldrich) overnight at 4°C. Immunoprecipitated proteins were analysed by immunoblotting as described above using mouse anti-HA (1/1000, Covance, MMS-101R), rabbit anti-5-HT6R antibody (1/500, Abcam, ab103016) and an anti-Cdk5 antibody (1/500, Cell Signaling, #2506), and quantified in four independent experiments, as described above.

### *In vivo* proliferation index

For the E15.5 proliferation index, *in utero* electroporation was performed at E14.5 with either 5-HT6R-shRNA1 and pUB6-tdTomato (*n*=6 brains) or scram-shRNA and pUB6-tdTomato (*n*=4 brains); bromodeoxyuridine (BrdU) (50 mg/kg, Sigma) was injected intraperitoneally at E15.5 and dams were sacrificed 4 h later. Brains from embryos were extracted and coronal brain sections were processed for BrdU labelling as previously described (Riccio et al., 2012).

### Determination of cAMP production

cAMP measurement was performed using Bioluminescence Resonance Energy Transfer (BRET) (Jiang et al., 2007): NG108-15 cells were co-transfected with 5-HT6R and the cAMP sensor CAMYEL constructs, and plated in white 96-well plates (Greiner) at a density of 80,000 cells per well. Twenty-four hours after transfection, cells were washed with PBS containing calcium and magnesium. Coelanterazine H (Molecular Probes) was added at a final concentration of 5 µM and left at room temperature for 5 min. BRET was measured using a Mithras LB 940 plate reader (Berthold Technologies).

### Time-lapse imaging in cortical slices

E17.5 acute brain slices were prepared as described previously (Riccio et al., 2011) from embryos electroporated at E14.5. Briefly brains were extracted and embedded in cold Hanks' balanced solution (HBSS, Invitrogen) with 3% ultra pure low melting point agarose (LMP agarose; Invitrogen or Roth). Slices (250 µm) were then cut on a Vibratome (VT1000S; Leica), placed on porous nitrocellulose filters (Millicell-CM, Millipore) in Fluorodishes (WPI) supplemented with NBM (Invitrogen) (Riccio et al., 2011). TOM+ PNs in cortical slices were imaged with an inverted confocal microscope (Nikon A1R) equipped for live imaging (Life Technologies) with long-working distance objectives (CFI Plan Fluor ELWD 20× C; NA: 0.45, Nikon and CFI Plan Fluor ELWD 40× C; NA: 0.60, Nikon). The microscope incubation chamber temperature was kept at 37°C with a constant flux (25 l/h) of 5% CO_2_ humidified at 96%. Stacks (50 µm; 3 µm stepped) were acquired every 10 min between the 10 and 15 h timepoints with resonant laser scanning to reduce toxicity and to avoid bleaching. The first 90-120 min of all movies were removed from analysis to avoid bias in measurements due to adaptation of the slice in the chamber incubator. Stacks were piled up using NIS-Elements (Nikon Software) to obtain orthogonal maximal projections that were then aligned using Metamorph (Molecular Devices, version 7.7.6). Piled stacks were orientated to align the direction of radial migration on the *y*-axis. For quantification of migration, speed scram-shRNA TOM+ PNs (*n*=98 cells), 5-HT6R-shRNA1 TOM+ PNs (*n*=94 cells) and Cdk5/p35+5-HT6R-shRNA1 TOM+ PNs (*n*=95 cells) located in deep cortical layers were tracked using Metamorph. Movements towards the pia were considered positive, whereas those towards ventricle were negative. Radial migration speed was calculated as the total distance travelled by the cell divided by total imaging time (minimum 4 h; maximum 6 h). For quantification of the multipolar-bipolar transition at the border between the IZ and deep cortical layers, 280/100 µm boxes with the upper border aligned on the IZ/deep layers boundary were drawn. All multipolar-shaped cells contained in this box were tracked and the percentage of cells transiting from a multipolar-like morphology to a bipolar migration stage was calculated during an 8 h time period. Scram-shRNA TOM+ PNs (*n*=76 cells), 5-HT6R-shRNA1 TOM+ PNs (*n*=79 cells) and Cdk5/p35+5-HT6R-shRNA1 TOM+ PNs (*n*=85 cells) were tracked. For live-imaging experiments, electroporated slices in each experimental condition were obtained from brains following *in utero* electroporation of three independent dams.

### *In vivo* quantification

Images were acquired using an epifluorescence microscope (Nikon Eclipse 90i) equipped with a 10× objective (Plan Apo 10×/0.45, NA: 1, Nikon) or a confocal (Zeiss LSM700) microscope equipped with a dry 10× objective (Plan-Neofluar 10×/0.30, Zeiss), a 20× objective (Plan-Neofluar 20×/0.50, Zeiss) and an oil-immersion 40× objective (Plan-Neofluar 40×/1.3 Oil, Zeiss). Images from coronal sections of 5-HT6R-shRNA1 (*n*=6 brains), 5-HT6R-shRNA2 (*n*=4 brains), scram-shRNA (*n*=6 brains), Cdk5/p35 rescue (*n*=7 brains), (h)5-HT6R rescue (*n*=7 brains), (h)5-HT6R-Gs dead rescue (*n*=6 brains), (h)5-HT6R-D106A rescue (*n*=5 brains), NeuroD-Cre-IRES-EGFP (*n*=4 brains), NeuroD-Cre-IRES-EGFP; floxed 5-HT6R-shRNA1 (*n*=5 brains) and (h)-5HT6R overexpression (*n*=8 brains) were obtained at P19.0 following *in utero* electroporation of constructs at E14.5. Electroporated embryos were obtained from at least three independent dams. Distribution of cells was quantified by apposing a 12-bin grid at the level of the somatosensory cortex. Bins corresponding to the CP, deep cortical layers and intermediate zone were pooled. At P7 the number of misplaced 5-HT6R-shRNA1 TOM+ cells (*n*=5 brains) and scram-shRNA1 TOM+ cells (*n*=6 brains) in layers 5 and 6 were counted per region of interest in the somatosensory cortex. Quantification of neuronal differentiation markers was performed using confocal microscopy and the percentage of 5-HT6R-shRNA1 TOM+ cells and scram-shRNA TOM+ cells expressing BrdU (1166 cells, *n*=5 brains, 5-HT6R-shRNA1; 968 cells, *n*=4 brains, scram-shRNA), TBR1 (1361 cells, *n*=2 brains, 5-HT6R-shRNA1; 1341 cells, *n*=2 brains, scram-shRNA), TBR2 (310 cells, *n*=3 brains, 5-HT6R-shRNA1; 389 cells, *n*=4 brains, scram-shRNA), Ngn2 (971 cells, *n*=2 brains, 5-HT6R-shRNA1; 770 cells, *n*=2 brains, scram-shRNA), Brn2 (1556 cells, *n*=2 brains, 5-HT6R-shRNA1; 1026 cells, *n*=2 brains scram-shRNA), SATB2 (1577 cells, *n*=2 brains, 5-HT6R-shRNA1; 1444 cells, *n*=2 brains, scram-shRNA) and CUX1 (406 cells, *n*=5 brains, 5-HT6R-shRNA; 701 cells, *n*=7 brains, scram-shRNA) were analysed.

### Statistical analysis

Statistical analysis (GraphPad Prism software, version 6.0) was performed using an unpaired Student's *t*-test or one-way analysis of variance with Tukey's multiple comparisons test.

## Supplementary Material

Supplementary Material
