# Supplementary material for: The serotonin 6 receptor controls neuronal migration during corticogenesis via a ligand-independent Cdk5-dependent mechanism
Source: Development. 2014 Sep;141(17):3370–7. doi: 10.1242/dev.108043 (PMC4199128; doi:10.1242/dev.108043)
Supplement: Supplementary Material [file supp_141_17_3370__index.html]

The serotonin 6 receptor controls neuronal migration during corticogenesis via a ligand-independent Cdk5-dependent mechanism — Supplementary Material 

# The serotonin 6 receptor controls neuronal migration during corticogenesis via a ligand-independent Cdk5-dependent mechanism

## DEV108043 Supplementary Material

**Files in this Data Supplement:**

- **Supplementary Material**
