# Supplementary material for: The serotonin 6 receptor controls neuronal migration during corticogenesis via a ligand-independent Cdk5-dependent mechanism
Source: Development. 2014 Sep;141(17):3370–7. doi: 10.1242/dev.108043 (PMC4199128; doi:10.1242/dev.108043)
Supplement: Supplementary Material [file supp_141.17.3370_DEV108043.pdf]

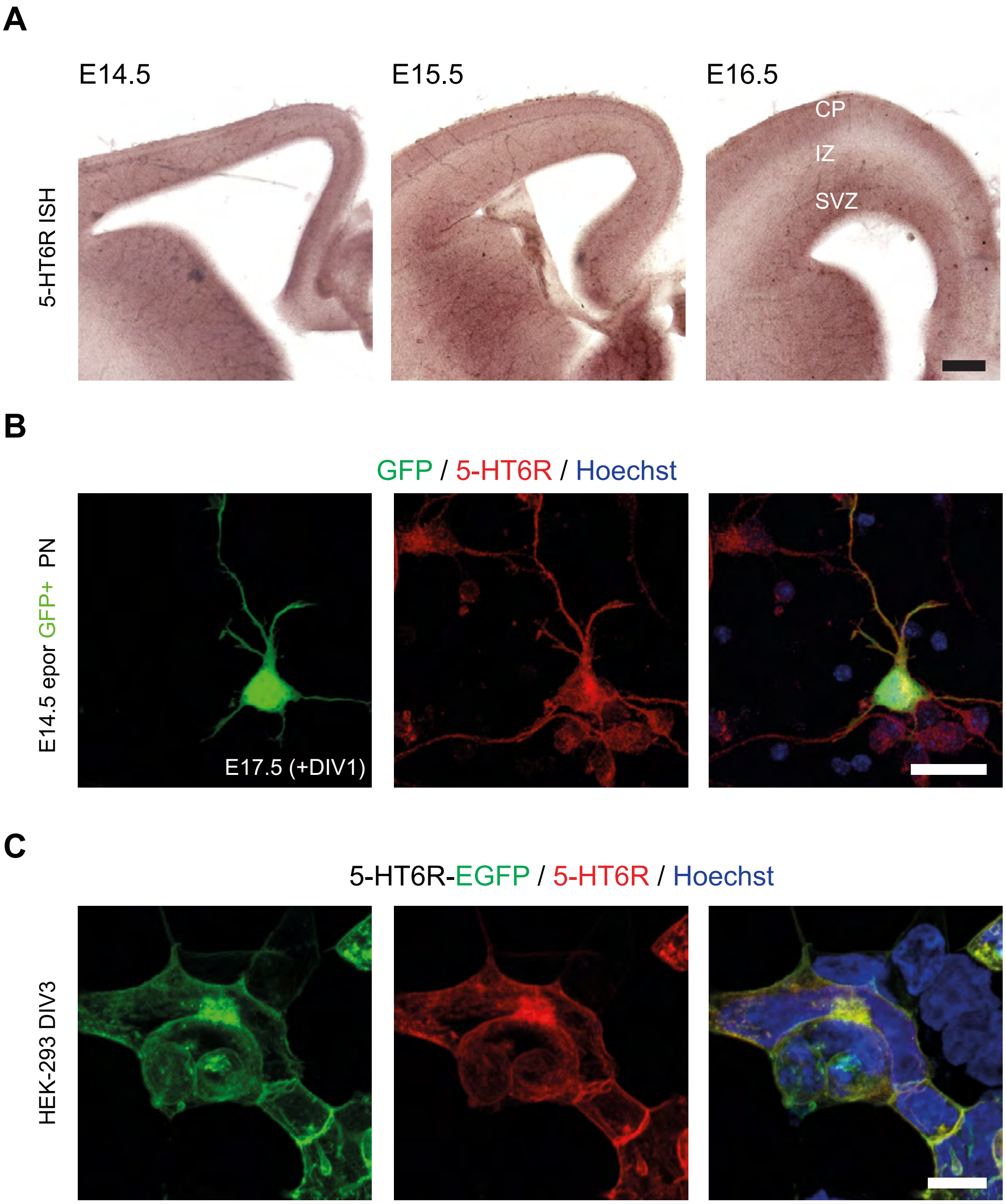

Supplementary Figure 1. (A) *In-situ* hybridization (ISH) time-course between E14.5 to E16.5 showing that 5-HT6R is broadly expressed in the SVZ, IZ and CP of the developing pallium. (B) Confocal images showing that the 5-HT6R is expressed in a TOM+ pyramidal neuron (PN). Cortical cultures were prepared from E17.5 cortices containing GFP+ PNs labeled following *in utero* electroporation (epor) at E14.5 and immunostained at day *in vitro* 1 (DIV1) with the 5-HT6R antibody. (C) The 5-HT6R antibody specifically labels HEK-293 cells transfected with a 5-HT6R GFP-tagged construct. Note that HEK-293 cells that do not contain the 5-HT6R-EGFP construct are not immunolabeled for the 5-HT6R. SVZ: subventricular zone, IZ: intermediate zone, CP: cortical plate. Scale bars: (A) 200 µm, (B) 15 µm , (C) 10 µm.

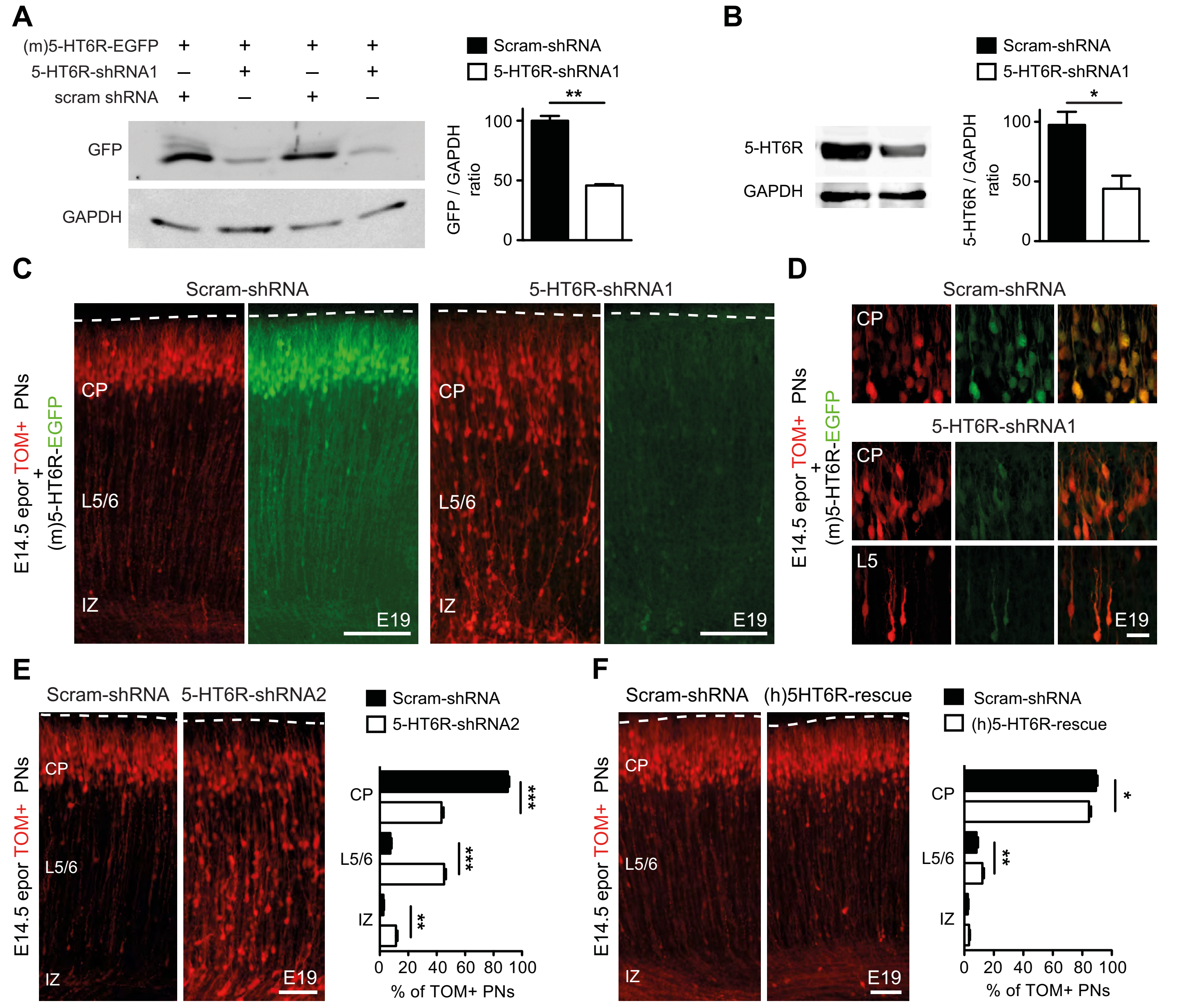

Supplementary Figure 2. (A) Western blots showing that 5-HT6R-shRNA1 significantly down-regulates expression of (m)5-HT6R-EGFP in HEK-293 cells (** $P < 0.01$, unpaired Student's t-test). (B) Western blots showing that 5-HT6R-shRNA1 significantly down-regulates expression of the endogenous 5-HT6Rs in primary cortical cultures. Neuronal cultures prepared from E14.5 cortices were nucleofected with 5-HT6R-shRNA1 or scram-shRNA and Western blots were performed on cell lysates at day *in vitro* 3. (* $P < 0.01$, unpaired Student's t-test). (C-D) At E19, 5-HT6R-shRNA1 down-regulates expression of (m)5-HT6R-EGFP following *in utero* electroporation (epor) at E14.5 compared to the scram-shRNA. Note residual low expression of 5-HT6R-EGFP following 5-HT6R knockdown (E) *In utero* epor of 5-HT6R-shRNA2 induces a significant mispositioning of PNs in the IZ, layer 5/6 and CP compared to scram-shRNA (*** $P < 0.001$, unpaired Student's t-test). (F) Over-expression of the (h)5-HT6R induces only a minor mispositioning of PNs at E19 following *in utero* epor at E14.5 (* $P < 0.05$, ** $P < 0.01$ unpaired Student's t-test). PN: pyramidal neuron, SVZ: subventricular zone, IZ: intermediate zone, CP: cortical plate. Error bars are means ± s.e.m. Scale bars: (C, E, F) 100 µm, (D) 20 µm.

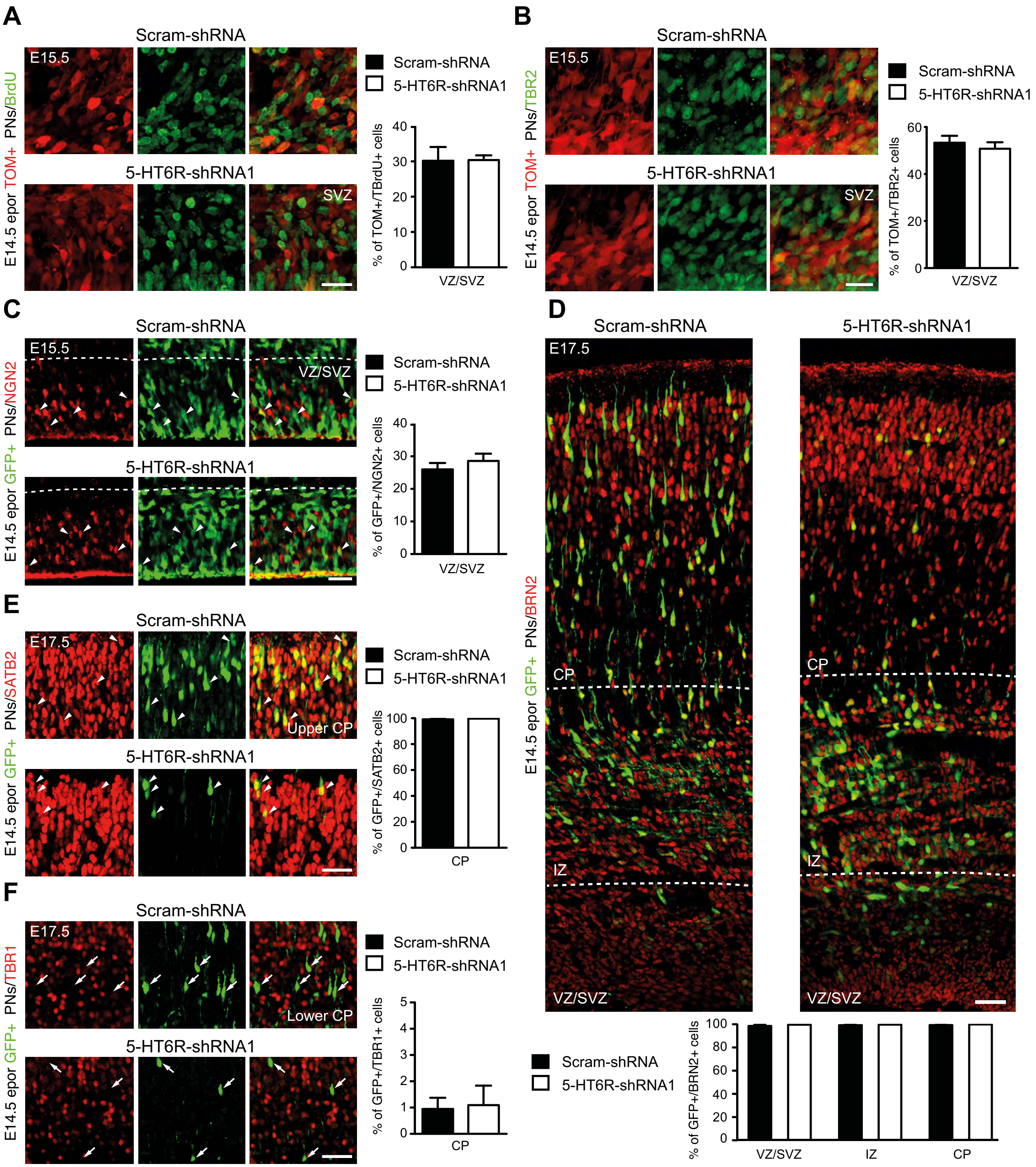

Supplementary Figure 3. 5-HT6R knockdown does not affect early steps of proliferation and neuronal differentiation. (A) BrdU proliferation index at E15.5 did not show any significant differences between the percentage of 5-HT6R-shRNA TOM+ PNs colabeled with BrdU compared to the scram-shRNA condition (B-D) Immunohistochemistry (IHC) for early regulators of neuronal differentiation did not reveal any significant differences in the percentage of HT6R-shRNA1 *vs.* scram-shRNA TOM+ PNs colabeled with TBR2 at E15.5 (B), Neurogenin2 (NGN2) at E15.5 (C) and the POU-III transcription factor BRN2 at E17.5. (D) (E-F) IHC did not reveal any significant differences in the percentage of 5-HT6R-shRNA1 *vs.* scram-shRNA TOM+ PNs colabeled with SATB2, a marker of superficial-layer molecular identity or TBR1, a marker of deep-layer molecular identity. Arrowheads indicate co-localized cells. Arrows show electroporated cells that do not co-localize with TBR1. PNs: pyramidal neurons, VZ: ventricular zone, SVZ: subventricular zone, IZ: intermediate zone, CP: cortical plate. Error bars are means ± s.e.m. Scale bars: (A, B) 20 µm, (C-F) 50 µm.

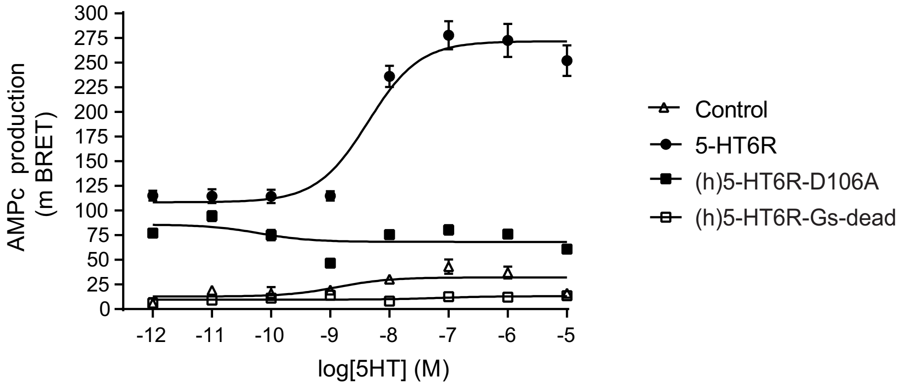

Supplementary Figure 4. Cyclic AMP production in transfected NG108-15 cells showing that the (h)5-HT6R-Gs-dead mutant does not elicit cAMP production in absence or presence of 5-HT compared to the wild-type (h)5-HT6R. The (h)5-HT6R-D106A mutant displays constitutive cAMP production, which is not further enhanced by 5-HT. Control condition represents cAMP production in cells transfected with empty plasmid. 5HT: serotonin.

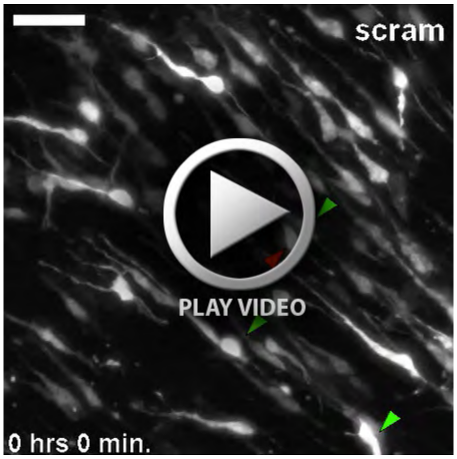

Supplementary Movie 1. Time-lapse sequence showing that E17.5 scram-shRNA TOM+ electroporated pyramidal neurons migrate radially in deep cortical layers. A majority of neurons exhibit a well-defined bipolar morphology and migrate continuously. Green arrowheads indicate cells that migrate radially whereas red arrowheads indicate cells that remain stationary during the time-lapse sequence. Scale bar: 50 μm.

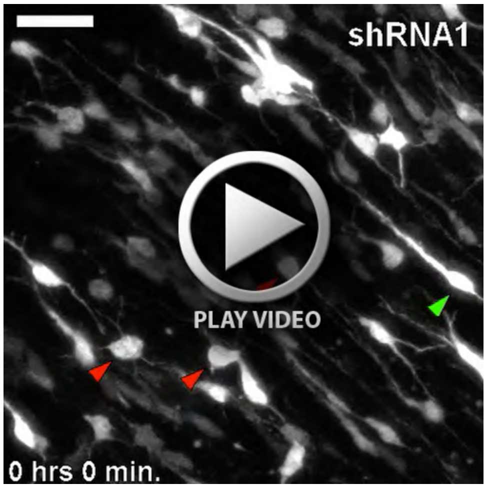

Supplementary Movie 2. Time-lapse sequence showing that E17.5 5-HT6R-shRNA1 TOM+ electroporated pyramidal neurons migrate abnormally in deep cortical layers. A majority of neurons do not display a normal migratory bipolar morphology and remain stationary. Red arrowheads indicate cells that remain stationary during the time-lapse sequence and display an abnormal multipolar morphology whereas green arrowheads indicate cells that migrate radially. Scale bar: 50 µm.

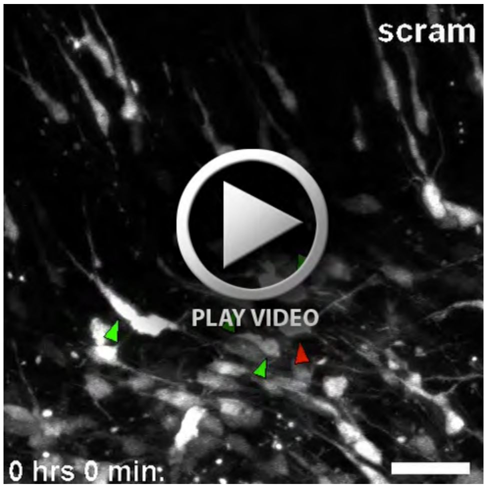

Supplementary Movie 3: Time-lapse sequence showing that E17.5 scram-shRNA TOM+ electroporated pyramidal neurons switch from a multipolar morphology in the intermediate zone (IZ) to a bipolar mode of radial migration in deep cortical layers. Green arrowheads indicate multipolar cells in IZ that switch to radial migration (green arrows). Red arrowheads indicate multipolar cells that do not switch to radial migration during the time-lapse sequence. Scale bar: 50 µm.

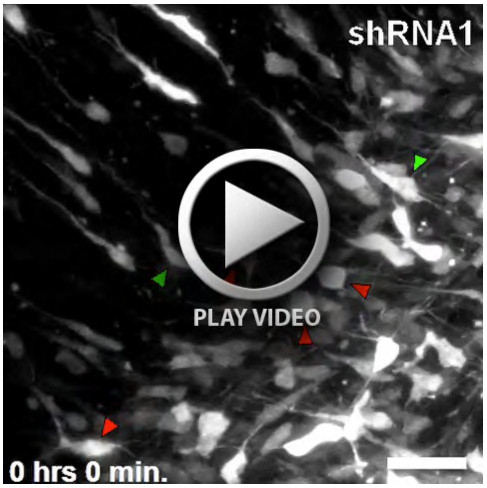

Supplementary Movie 4: Time-lapse sequence showing that a majority of E17.5 5-HT6R-shRNA1 TOM+ electroporated pyramidal neurons fail to switch from a multipolar morphology in the intermediate zone (IZ) to a bipolar mode of radial migration in deep cortical layers. Red arrowheads indicate multipolar cells that do not switch to radial migration during the time-lapse sequence. Green arrowheads indicate multipolar cells that switch to radial migration (green arrows). Note that TOM+ neurons switching to radial migration can present abnormal backward movement towards the IZ. Scale bar: 50 µm.

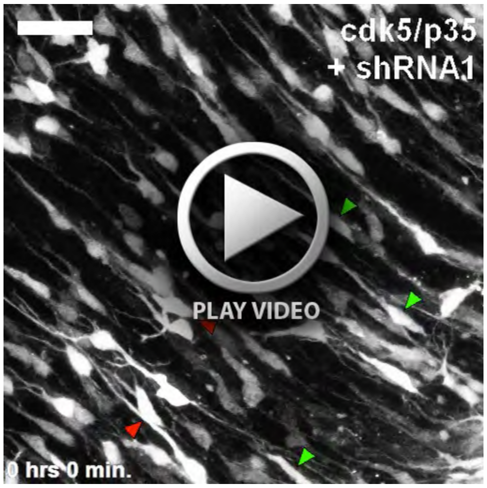

Supplementary Movie 5. Time-lapse sequence showing that Cdk5/p35 over-expression in E17.5 5-HT6R-shRNA1 TOM+ electroporated pyramidal neurons rescues radial migration in deep cortical layers. Green arrowheads indicate cells that migrate radially whereas red arrowheads indicate cells that remain stationary during the time-lapse sequence. Scale bar: 50 µm.

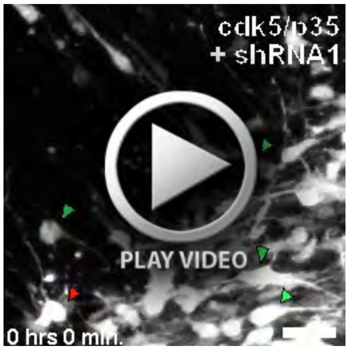

Supplementary Movie 6. Time-lapse sequence showing that Cdk5/p35 over-expression in E17.5 5-HT6R-shRNA1 TOM+ electroporated pyramidal neurons rescues the switch from a multipolar morphology in the intermediate zone (IZ) to a bipolar mode of radial migration in deep cortical layers. Green arrowheads indicate multipolar cells in IZ that switch to radial migration (green arrows). Red arrowheads indicate multipolar cells that do not switch to radial migration during the time-lapse sequence. Scale bar: 50 µm.
